# The relationship between frailty status and psychosocial indices in older Korean adults

**DOI:** 10.1007/s11357-025-02041-6

**Published:** 2025-12-17

**Authors:** Ji Young Kim, Taesic Lee, Sangbaek Koh

**Affiliations:** 1 Department of Family Medicine, Yonsei University Wonju College of Medicine, Wonju, Korea; 2 Department of Preventive Medicine, Yonsei University Wonju College of Medicine, Wonju, Korea; 3 Department of Convergence Medicine, Yonsei University Wonju College of Medicine, Wonju, Korea

**Keywords:** Older Korean cohort, ARIRANG cohort, Aging-cognition cohort, Frailty, Psychosocial factors

## Abstract

Frailty is a heightened vulnerability to physiological stressors caused by cumulative physiological decline over a lifespan. Psychological issues (depression and stress) and social problems (such as loneliness and social isolation) serve as potential risk factors for unfavorable comorbidities, including frailty, cardiometabolic disorders, and unexpected mortality. In this study, we aimed to elucidate the relationship between frailty and psychosocial factors in an aging population. The Aging-Cognition Cohort, a subset of the Wonju-Pyeongchang Arirang Cohort, included 930 participants aged 55–79 between 2020 and 2022. Participants underwent a frailty assessment based on Fried’s criteria. Pearson’s method-based network analysis identified key factors among five psychosocial indices (PSIs). Univariate and multivariate regression (linear and logistic methods) analyses were used to explore the relationship between frailty and the five PSIs. Network analysis revealed differential connectivity between the five PSIs and clinical biomarkers across different frailty conditions. Among psychosocial factors, the University of California Los Angeles (UCLA) Loneliness Scale emerged as a key indicator. The association of summarized values of the five PSIs (eigenvalue) and UCLA with frailty exhibited significant results independent of the covariates (eigenvalue: beta-coefficient (B) = −0.018, 95% confidence interval (CI) −0.022–0.014; UCLA: B = 0.018, 95% CI 0.012–0.024). The relationship between PSIs and frailty was maintained across all subgroup analyses. This study highlights the robust association between frailty and psychosocial factors in older Korean population.

**Supplementary information:**

The online version contains supplementary material available at https://doi.org/10.1007/s11357-025-02041-6.

## Introduction

Frailty is a clinical syndrome that represents a state of increased vulnerability to difficulties in restoring homeostasis after a stressful event, resulting from the cumulative decline of physiological systems over a lifetime [1]. Frailty becomes highly prevalent with increasing age; thus, its prevalence is anticipated to increase in tandem with the increasing average life expectancy of the population [2]. Being frail elevates an individual’s susceptibility to adverse outcomes, including the loss of activities of daily living, physical limitations, falls, hospitalization, and mortality [3, 4]. Consequently, the identification of frailty through assessment and implementation of intervention strategies aimed at reducing and preventing it has become a crucial topic among the older adults.

One of the most widely accepted frameworks for assessing and defining physical frailty is Fried’s criteria, which systematically capture various components contributing to frailty [5]. Developed by Dr. Linda P. Fried in 2001, this criteria serve as a comprehensive and validated framework for identifying and characterizing physical frailty. Fried’s criteria assess frailty by evaluating five key characteristics: unintentional weight loss, weakness, poor endurance (exhaustion), slowness, and low activity [5]. As defined by Fried’s criteria, physical frailty is associated with a significantly elevated risk of adverse health outcomes in older adults. However, psychological and social aspects are often overlooked.

Psychological problems, such as depression and stress, and social problems, such as loneliness and social isolation, are both related to negative health outcomes. Loneliness in the older adults is a significant risk factor for physical health problems and psychiatric conditions, such as depression and cognitive impairment [6]. Moreover, older individuals with increased loneliness and social isolation are at an increased risk of developing physical frailty [7, 8]. Geriatric psychological problems such as depression and stress enhance the incidence of frailty, worsen the outcomes of chronic medical illnesses such as cardiovascular diseases, neurologic disorders, and cancer, and even increase mortality [9–11].

Despite being distinct constructs, frailty and psychosocial problems often coexist [7, 11]. However, the relationships among these constructs remains complex and not fully understood. In this study, we examined the association between frailty and various psychosocial indices (PSIs), including depressive symptoms, stress, social support, and loneliness, using data from a cross-sectional cohort of community-dwelling older Korean adults. Specifically, we employed validated measures such as the Psychological Well-Being Index (PWI), Center for Epidemiologic Studies Depression Scale (CES-D), Geriatric Depression Scale (GDS), Multidimensional Scale of Perceived Social Support (MSPSS), and University of California, Los Angeles (UCLA) Loneliness Scale. Understanding this relationship is vital for providing comprehensive care to aging populations and for enhancing our understanding of the intricate dynamics of aging and frailty.

## Methods

### Study population and data collection

This study was conducted using the Aging-Cognition Cohort, a sub-cohort of the Korean Genome and Epidemiology Study on Atherosclerosis Risk of Rural Areas in the Korean General Population (KoGES-ARIRANG). KoGES-ARIRANG is a population-based prospective cohort study aimed at assessing the prevalence, incidence, and risk factors for chronic degenerative disorders [12, 13]. The Aging-Cognition Cohort is a population-based prospective cohort study that assessed the frailty and cognitive status of Korean adults aged 55–79 years. It consisted of general demographic factors, physical and blood tests, and multiple questionnaires. The data comprise three sub-datasets established annually from 2020 to 2022. The clinical variables were preprocessed by unifying clinical features and eliminating missing values, and then integrated into a single large dataset (*N* = 926, Fig. S1).

### Frailty score

Frailty scores were calculated based on Fried’s criteria, ranging from 0 to 5 points [5]. Fried’s criteria calculate frailty status based on the following five criteria: weight loss, weakness, exhaustion, slowness, and low activity. A score of 0 points corresponds to robust, 1–2 points to prefrail, and 3–5 points to frail. The specific conditions for each criterion are explained in Supplementary Data (Table S1) [5, 14].

### Psychosocial indices

We selected the following five PSIs related to frailty based on literature review and data availability: PWI, CES, GDS, UCLA, and MSPSS (Fig. S2). Five psychosocial indices, including three psychological and two social measures, were used to evaluate their associations with frailty (Table S2). Among the psychological indices, stress was assessed using the PWI [15]. The PWI comprises 18 items, each scored from 0 to 3, yielding a total score ranging from 0 to 54, with higher scores indicating higher levels of stress. Depressive symptoms were evaluated using the CES and GDS. The CES consists of 20 items, each scored from 1 to 4, with a total score ranging from 20 to 80; higher scores indicate higher levels of depressive symptoms [16]. The GDS includes 30 items, each scored as either 0 or 1, with a maximum score of 30; higher GDS scores indicated more severe depressive symptoms [17].

For social indices, loneliness was measured using the UCLA Loneliness Scale [18], which comprises 20 items, each rated from 1 to 4, resulting in a total score range of 20–80, with higher scores reflecting greater loneliness. Social support was assessed using the MSPSS [19], which contains 12 items, each scored from 1 to 5. The total score ranged from 12 to 60, with higher scores indicating greater perceived social support.

### Statistical analysis

**Fig. 1:**
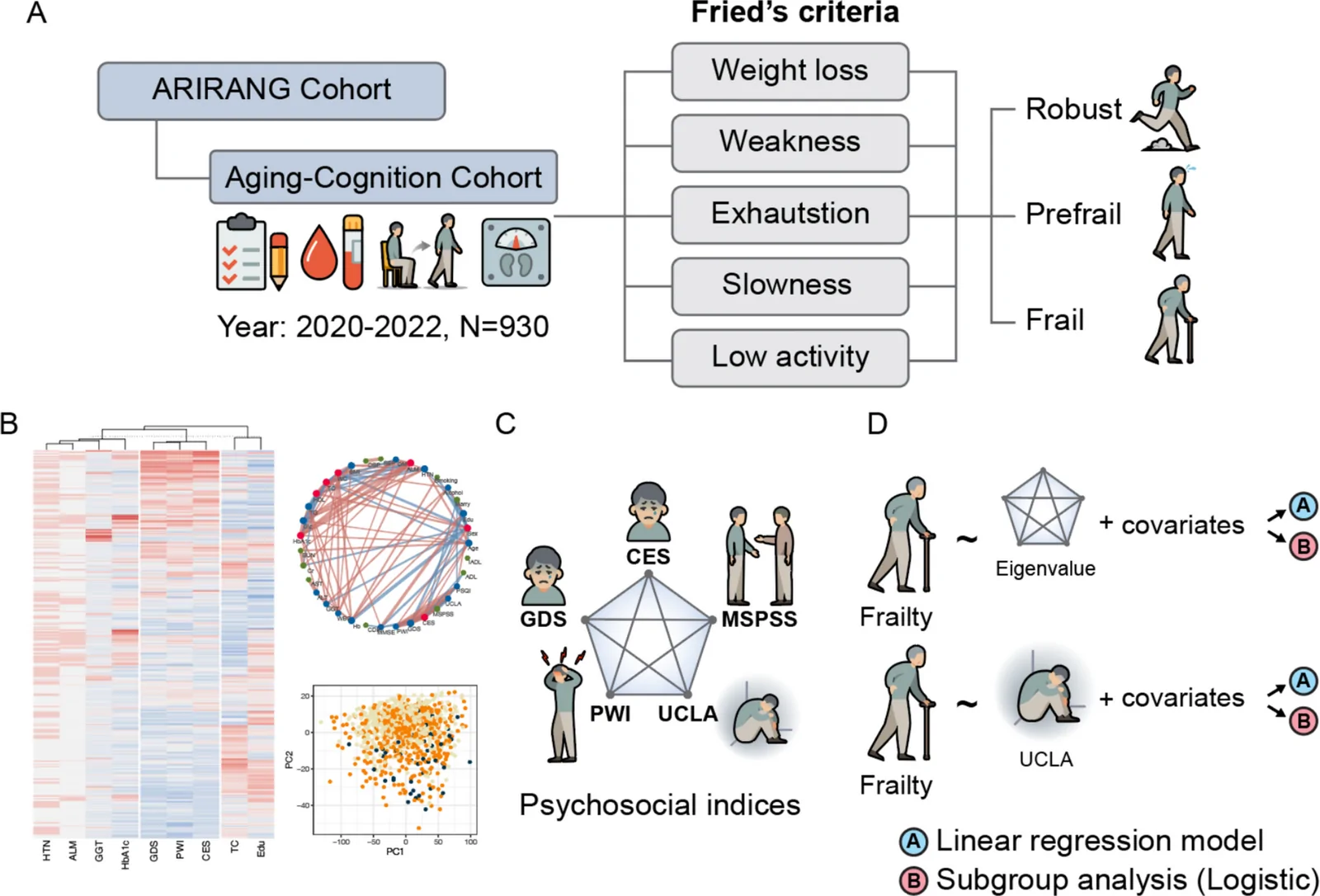
Study design for identifying the associations between PSIs and frailty. This study aimed to investigate the associations between PSIs such as stress, depression, and loneliness with frailty in older adults. Five key analytical activities were undertaken as part of the research: **A** The Aging-Cognition Cohort, derived from the Wonju-Pyeongchang Arirang Cohort, comprised three annual datasets. Standardization of variables was performed across these datasets to ensure consistency, and subjects with missing data were excluded. The resulting datasets were merged to form a comprehensive dataset. The cohort includes quantitative measures of weight loss, weakness, exhaustion, slowness, and low activity. Using Fried’s frailty criteria, participants were classified into three groups: robust, prefrail, and frail, based on a frailty score derived from these five indices. **B** An unsupervised analytical module, featuring clustering algorithms, network analysis, and dimensionality reduction techniques, was employed to identify global signatures across clinical, laboratory, and psychosocial variables. **C** The interrelationships among the five PSIs were analyzed to identify the most representative indicator. **D** The relationships between the representative PSIs (UCLA and eigenvalue) and the frailty score were analyzed using linear regression. Finally, a subgroup analysis of the previously identified associations was conducted through logistic regression. GDS, Geriatric Depression Scale; CES, Center for Epidemiologic Studies Depression Scale; MSPSS, Multidimensional Scale of Perceived Social Support; UCLA, University of California, Los Angeles Loneliness Scale; PWI, Psychological Well-Being Index; PSI, psychosocial index

The overall study design is displayed in Fig. 1. We employed both supervised and unsupervised approaches to understand the overall characteristics and structure of the data. Firstly, a supervised statistical approach was applied, where frailty status (robust, pre-frail, and frail) served as the outcome variable, and the general cohort characteristics were examined using Chi-square tests for categorical variables and ANOVA for continuous variables (Table S3).

We constructed PCC-based networks for each frailty state to identify whether any variables or variable modules exhibited disorder-related changes in network structure, such as reduced connectivity or rewiring. Pearson correlation matrices were constructed for the robust, prefrail, and frail groups. A network-specific cutoff was selected to best highlight group-wise pattern changes while balancing interpretability and network fragmentation**.** Variables with the top 50% connectivity in the network analysis were extracted separately for each group, and a Venn diagram was generated to display the overlapping variables.

Next, hierarchical clustering was performed on the variables that ranked in the top 50% across all three groups. In detail, we performed the hierarchical clustering using Euclidean distance and the complete linkage method. These variables were subjected to principal component analysis (PCA) for dimensionality reduction.

Univariate association analyses were performed to assess the relationships between frailty and various clinical, laboratory, and psychosocial variables (Table S4). The frailty score, ranging from 0 to 5, was transformed into four distinct formats: two continuous and two categorical. Variables that consistently showed significant associations across multiple frailty forms (*p* < 0.05) were specified.

Several analyses were conducted to identify the most representative index of the five PSIs. First, the eigenvalues of the five PSIs were calculated using principal component analysis, with the PC1 value adopted as the representative eigenvalue for the five PSIs. A 5 × 5 correlation matrix was constructed using both Pearson and Spearman methods to elucidate the interrelations between the five PSIs. Hierarchical clustering and PCA were subsequently applied to further explore the relationships between these indices and the other variables. Subsequently, a network analysis of the PSI correlations was conducted based on the PCCs to identify the hub index among the five indices.

The association between the representative PSI eigenvalue and UCLA score was examined using linear regression analysis. First, the distribution of eigenvalues and UCLA scores based on the frailty scores was analyzed. Subsequently, multivariate analysis was employed for this investigation, with covariates selected based on previously established combinations from previous studies (Table S5) [20–23]. The three models were applied in a stepwise manner.

Finally, subgroup analysis was conducted to explore the relationship between frailty score and PSIs across different subgroups, including age, gender, education, Instrumental Activities of Daily Living (IADL), and Pittsburgh Sleep Quality Index (PSQI). Subgroup analysis was performed using multivariate logistic regression by applying the third model from the linear regression analysis, which included all covariates.

Data were analyzed using R (version 4.3.3), a statistical computing and graphics software environment. Descriptive statistics were generated for baseline characteristics and inferential statistical tests were performed to evaluate the associations between psychosocial indices and frailty. Statistical significance was set at *p* < 0.05.

## Results

The Aging-Cognition cohort comprised 930 participants with a mean age of 68 years, ranging from 55 to 79 years. Frailty was assessed using the Fried frailty criteria with scores ranging from 0 to 5 (Table S3). Participants were categorized into three groups based on their frailty scores: robust (score 0), prefrail (1–2 points), and frail (3–5 points). The distribution of the frailty scores followed a left-skewed pattern, with lower scores being more common (Fig. S3A). The robust and prefrail groups had similar proportions, whereas the frail group represented only 5% of the total cohort (Fig. S3B).

We analyzed the linear trends of the clinical and laboratory variables according to the three frailty groups (Table S3). As stepwise increments from robust, prefrail, and frail, the following clinical, medical, and laboratory variables exhibited significant linear trends: age, education, alcohol consumption, diabetes mellitus (DM), dyslipidemia (anti-lipid medication, ALM), total cholesterol (TC), high-density lipoprotein (HDL), hemoglobin A1c (HbA1c), and hemoglobin (Hb). PSIs (MMSE, CDR, PWI, GDS, CES, MSPSS, UCLA, PSQI, ADL, and IADL) showed significant correlations with worsening frailty status.

**Fig. 2:**
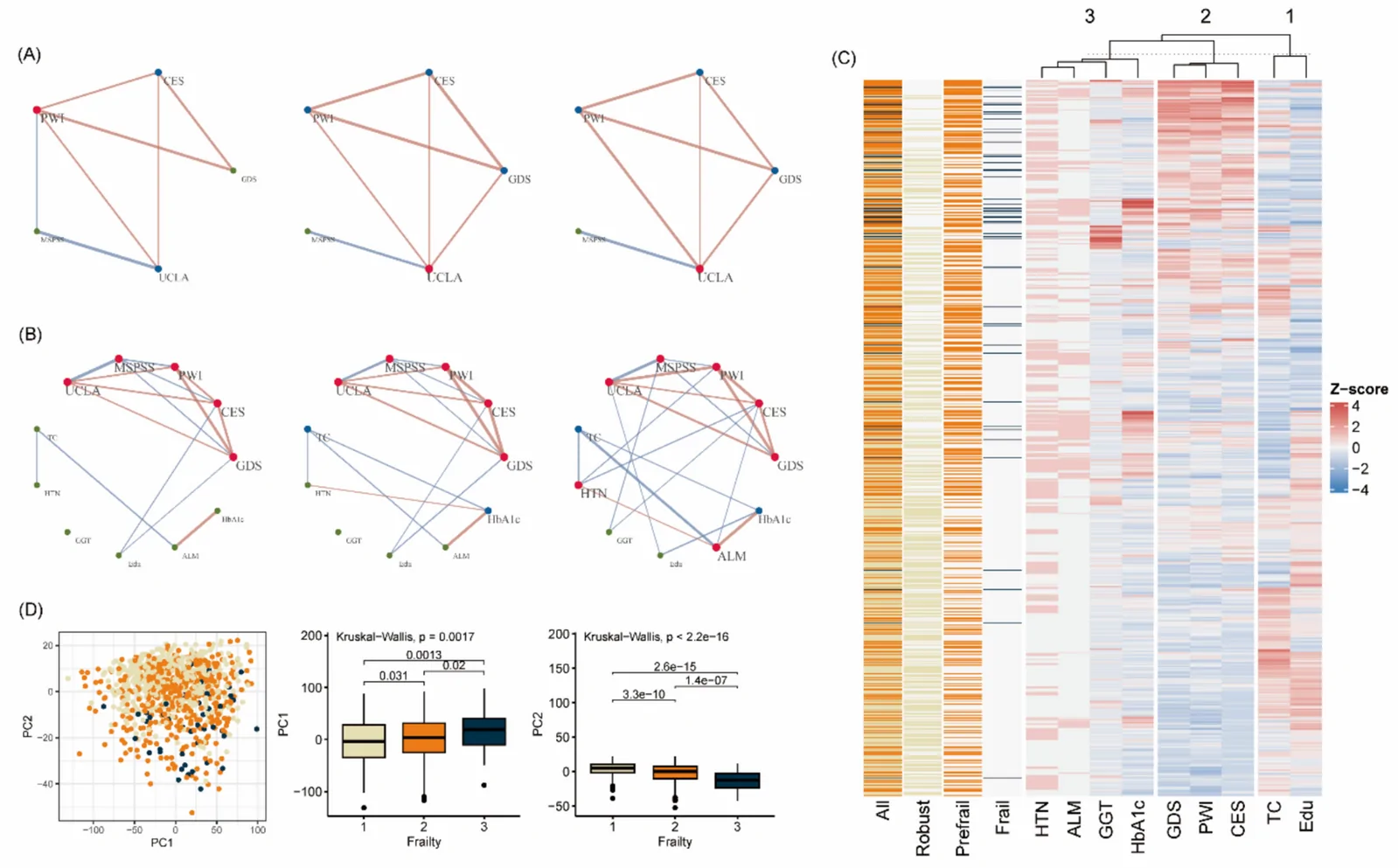
Network patterns of PSIs and hub features across frailty, and multivariate profiling. **A** Networks among psychosocial indices (PSIs: GDS, CES, PWI, MSPSS, UCLA) stratified by frailty status (robust, prefrail, frail). Nodes represent PSIs; edges depict Pearson correlation coefficients (PCCs) with a threshold of |PCC|≥ 0.35. Edge color encodes correlation direction (red, positive; blue, negative) and thickness is proportional to |PCC|. **B** Integrated networks of PSIs with hub variables (clinical/laboratory features exhibiting high connectivity in the global feature network; refer to Figs. S4 and S5 for identification procedure). Edges reflect PCCs with a threshold of |PCC|≥ 0.20. Layout, color scheme, and edge weighting are as in panel **A**. **C** Hierarchical clustering of frailty strata through hub variables. Columns in the heatmap are the hub features, arranged into modules by hierarchical clustering (dendrogram above). Rows represent subjects ordered by the hierarchical clustering method. The four vertical bars to the left of the heatmap display frailty status (all, robust, prefrail, frail). **D** Left, scatter plot of individuals embedded by a dimensionality-reduction of the hub variables using principal component analysis. Point colors indicate frailty status (robust as yellow, prefrail as orange, and frail as blue). Right, boxplots showing the distributions of PC1 and PC2 scores across frailty strata (left to right: robust, prefrail, frail); Kruskal–Wallis *p*-values and pairwise comparisons are annotated above the plots. GDS, Geriatric Depression Scale; CES, Center for Epidemiologic Studies Depression Scale; PWI, Psychological Well-Being Index; MSPSS, Multidimensional Scale of Perceived Social Support; UCLA, University of California, Los Angeles Loneliness Scale; HTN, hypertension; ALM, anti-lipid medication; GGT, gamma-glutamyl transpeptidase; HbA1c, hemoglobin A1c; GDS, Geriatric Depression Scale; PWI, Psychological Well-Being Index; CES, Center for Epidemiologic Studies Depression Scale; TC, total cholesterol; Edu, education; PSI: psychosocial index

To characterize the connectivity patterns linking the five PSIs and clinical variables, we constructed group-specific networks for the robust, prefrail, and frail populations (Figs. 2, S4, and S5). Networks based solely on the five PSIs revealed that the frail network resembled the prefrail patterns, besides differing from the robust stage (Fig. 2A). According to t-statistics and the upper half of the degree (number of edges) distribution in the three networks, and based on their intersection, the following nine hub variables were identified: TC, HTN, GDS, GGT, CES, education, ALM, HbA1c, and PWI (Fig. S5). When the hub variables (*n* = 9) were incorporated into the PSI network at a PCC threshold of 0.30, the hubs clustered predominantly with other hubs and the PSIs with other PSIs, resulting in a fragmented network. To assess the interconnectivity between PSIs and clinical markers, the PCC cutoff was relaxed to 0.20, resulting that the frail and prefrail stages continued to exhibit comparable patterns, while the robust stage remained distinct (Fig. 2B).

To elucidate the modular structure of the nine hub features and the overall landscape of frailty-related characteristics, hierarchical clustering was conducted (Fig. 2C). Euclidean-based clustering revealed the clustering of stress (PWI) and sadness (GDS and CES), TC and educational level, and HTN and ALM (Fig. 2C). Additionally, cluster analysis stratified individuals into robust, prefrail, and frail groups (Fig. 2C). To gain an intuitive understanding of how individuals distribute across frailty states based on the hub variables, PCA was implemented, resulting in a clear classification of the robust, prefrail, and frail groups (Fig. 2D). In detail, comparison of PC1 and PC2 values showed that the differences between the frailty groups were statistically significant, with *p* < 0.05 for each pairwise comparison, indicating meaningful group differentiation. In additional experiments, when frailty-related variables were incorporated into PCA and clustering analyses, the degree of separation between frailty groups appeared to weaken (Fig. S6).

Individual associations between frailty and all variables were analyzed using linear regression (Fig. S7 and Table S4). Initially, the linear frailty score, which ranges from 0 to 5, was designated as the dependent variable, resulting in 23 clinical variables showing a significant relationship with the linear form of frailty. Subsequently, frailty was categorized into three stages—robust (1), prefrail (2), and frail (3)—transforming the frailty score into a categorical variable, which was then analyzed in relation to other variables. Most associations, except for white blood cell count and blood urea nitrogen level, showed trends similar to those of the linear frailty score. Treating frailty as a continuous variable demonstrated greater significance, with lower *p* values and larger beta values indicating a stronger association. In addition, the significantly related variables in categorical analysis were all included in the variables that are related in continuous analysis.

To further explore the relationships between frailty and associated variables, the frailty score was expanded from two categories to four distinct distributions using different thresholds (Fig. S8). In addition to the continuous and tertile forms of frailty, two binary forms were introduced into the circus plot: one dichotomized into robust and non-robust, and the other into frail and non-frail. Subsequent analysis of the relationships between these frailty distributions and clinical variables showed that age, education, alcohol consumption, ALM, TC, HDL, HbA1c, hemoglobin, CDR, MMSE, PWI, GDS, CES, MSPSS, UCLA, PSQI, and IADL were significantly associated with all forms of frailty, including both linear and binary models.

**Fig. 3:**
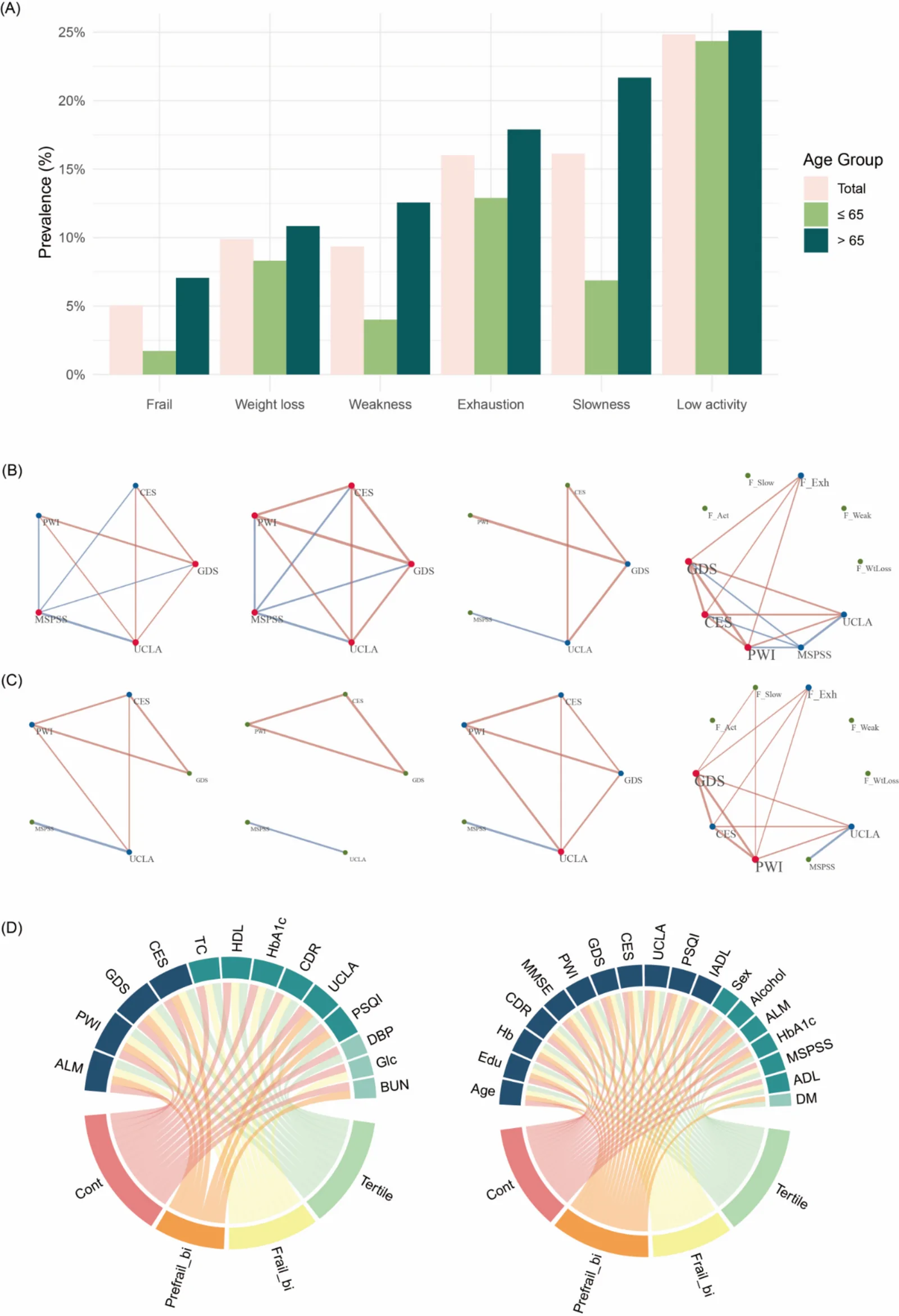
Age-stratified relationships between frailty and psychosocial/clinical variables. **A** Prevalence of overall frailty and of each frailty component (weight loss, weakness, exhaustion, slowness, and low activity) by age group (≤ 65 vs > 65 years). **B** Network patterns in participants aged ≤ 65 years. From left to right: pairwise networks among the five PSIs within the robust, prefrail, and frail strata (PCC cut off 0.35); far right, an integrated network linking PSIs with individual frailty components (PCC cut off 0.25). **C** Corresponding networks for participants aged > 65 years, shown in the same order as in **B**. For all networks, nodes represent variables and edges denote pairwise associations after thresholding; red edges indicate positive associations and blue edges negative associations; edge thickness reflects association magnitude. **D** Univariate associations between various frailty structures and psychosocial/clinical variables, visualized as circos plots (left, ≤ 65 years; right, > 65 years). PWI, Psychological Well-Being Index; GDS, Geriatric Depression Scale; CES, Center for Epidemiologic Studies Depression Scale; MSPSS, Multidimensional Scale of Perceived Social Support; UCLA, University of California, Los Angeles Loneliness Scale; F_Wtloss, weight loss; F_Weak, weakness; F_Exh, exhaustion; F_Slow, slowness; F_Act, low activity; ALM, anti-lipid medication; TC, total cholesterol; HDL, high-density lipoprotein; HbA1c, hemoglobin A1c; PSQI, Pittsburgh Sleep Quality Index; DBP, diastolic blood pressure; Glc, glucose; BUN, blood urea nitrogen; Edu, education; Hb, hemoglobin; MMSE, mini-mental state examination; IADL, instrumental activities of daily living; ADL, activities of daily living; DM, diabetes mellitus

To delineate age-related differences, we stratified participants into age of ≤ 65 and > 65 years and compared the prevalence of overall frailty and each component (weight loss, weakness, exhaustion, slowness, low activity). As expected, the prevalence of frailty showed a marked difference before and after the age of 65 (Fig. 3A). Although all five components showed high prevalence, two of them (weakness and slowness) exhibited particularly large differences between the two age groups, which likely account for much of the age difference in overall frailty burden (Fig. 3A). In PCC-based network analyses, the hub centrality of loneliness (UCLA) became more pronounced with increasing age. Among the five components, exhaustion and slowness showed the strongest and most consistent connections with the PSIs, more so in older group (Fig. 3B and C). In univariable analyses of frailty features with psychological and clinical variables, associations were more prominent in the older group than in the younger group (Fig. 3D).

The interaction among the five PSIs was examined through correlation matrices, hierarchical clustering, PCA, and network analysis (Figs. S9–S12). GDS, CES-D, and PWI showed high pairwise correlations (Pearson’s *r* ≥ 0.60) and clustered together in unsupervised analyses; notably, GDS and CES-D, both assessing depressive symptoms, were moderately correlated (*r* = 0.66). To address redundancy while preserving complementary information, these PSIs were analyzed alongside the others using dimensionality reduction (PCA) to derive a latent PSI score that mitigates collinearity. In the PCC-based network, loneliness (UCLA) served as a hub among the five PSIs (Fig. S9).

**Fig. 4:**
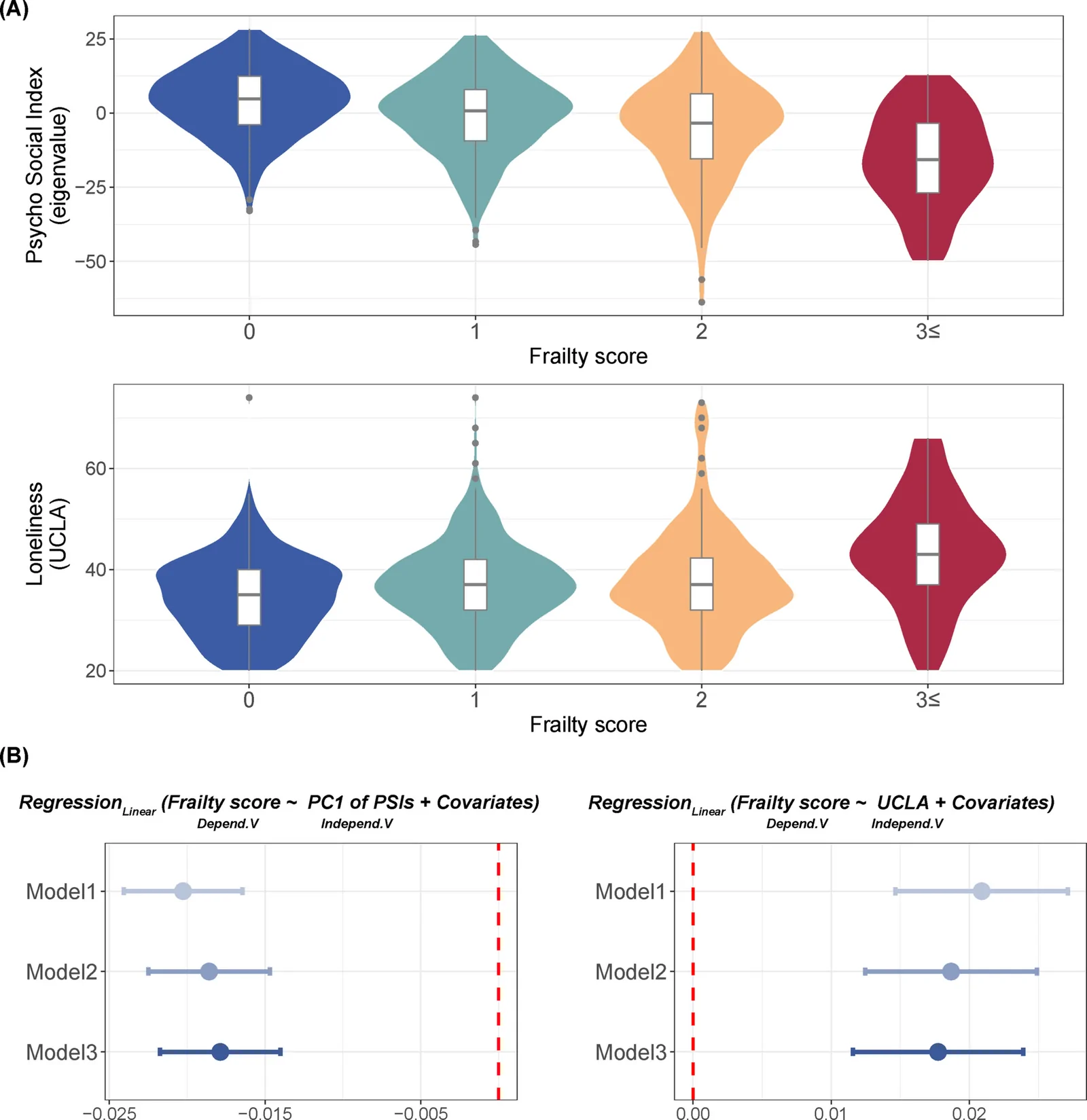
Association between frailty and PSIs. **A** Violin plots exhibit the distribution of PSI eigenvalue and loneliness (UCLA) scores according to frailty scores. The boxplots within the violin plots indicate the median and interquartile range of the eigenvalues and UCLA scores. **B** Multivariate linear regression models analyze the relationship between frailty and eigenvalues or UCLA scores. The models were adjusted for the following covariates; Model1: age, sex; Model2: education, marital status, alcohol, smoking, BMI; Model3: presence of disease (HTN, DM, HLD), and laboratory measures (TC, HDL, Hb). UCLA, University of California, Los Angeles Loneliness Scale; BMI, body mass index; HTN, hypertension; DM, diabetes mellitus; HLD, hyperlipidemia; TC, total cholesterol; HDL, high-density lipoprotein; Hb, hemoglobin; PSI, psychosocial index

Consequently, the eigenvalues of the five PSIs derived from PCA, and the UCLA loneliness score were selected as representative indicators of PSIs. The violin plot, illustrating the distribution according to frailty scores, showed that as the frailty score increased, the eigenvalue decreased, whereas the UCLA score increased (Fig. 4A). The association between frailty status and PSIs scores was analyzed using three multivariate models (Fig. 4B). The covariates included in each model were selected based on the variables implemented in prior studies and showed meaningful associations in this study (Table S5) [20–23]. Model 1 was adjusted for anthropometric factors. Model 2 included the covariates from Model 1, with the addition of lifestyle factors. Model 3 incorporated all the covariates from Model 2, along with the addition of chronic diseases and laboratory values. The association between loneliness (UCLA) and the representative PSI, specifically the eigenvalue derived from the PCA as the PC1 value of the five PSIs, with the frailty score, exhibited consistent significance across the three linear models (Fig. 4B, Tables S6 and S7); in linear analyses, the eigenvalue showed an inverse association with frailty (β =  − 0.020, 95% CI − 0.024 to − 0.016 in Model 1; − 0.019, − 0.023 to − 0.015 in Model 2; and − 0.018, − 0.022 to − 0.014 in Model 3), whereas loneliness showed a positive association with frailty (β = 0.021, 95% CI 0.015 to 0.027 in Model 1; 0.019, 0.013 to 0.025 in Model 2; and 0.018, 0.012 to 0.024 in Model 3). Furthermore, the association between binary frailty score—robust (frailty score 0) and non-robust (frailty score one or higher)—and loneliness (UCLA) was statistically significant level across all three models (Table S7). As seen in the earlier univariate regression, the linear form of frailty exhibited a stronger association with UCLA than dividing frailty into two groups, as evidenced by the lower *p*-values (Table S7).

**Fig. 5:**
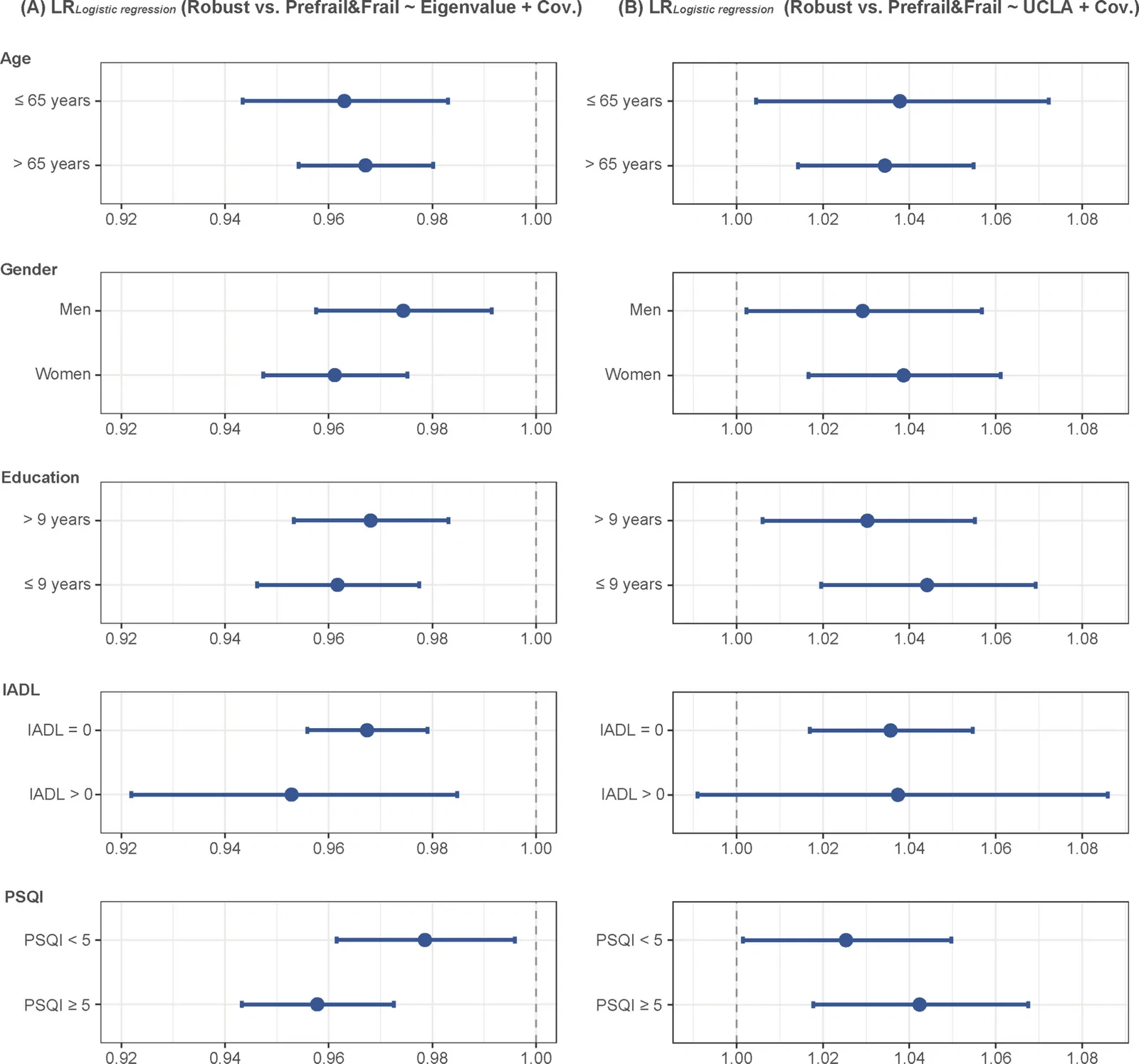
Subgroup analysis between frailty and PSI representatives: eigenvalue (left) or UCLA (right). The subgroup analyses were conducted according to age, gender, years of education, IADL score, and PSQI score. The logistic regression models compare the binary form of frailty (robust and non-robust) with Eigenvalue or UCLA, adjusted for the following covariates: age, sex, education, marital status, alcohol, smoking, BMI, presence of diseases (HTN, DM, HLD), and laboratory values (TC, HDL, Hb). PSI, psychosocial index; UCLA, University of California, Los Angeles Loneliness Scale; IALD, instrumental activities of daily living; PSQI, Pittsburgh Sleep Quality Index; HTN, hypertension; DM, diabetes mellitus; HLD, hyperlipidemia; TC, total cholesterol; HDL, high-density lipoprotein; Hb, hemoglobin

Finally, to assess whether the association between PSI and frailty persisted across the various subgroups, stratification analyses were conducted based on age, sex, and BMI (Fig. 5). Multivariate linear regression adjusted for all covariates from Model3 was used for subgroup analyses. The results revealed that the beta coefficients were positive across all subgroups, with statistically significant associations observed within the 95% confidence interval for all groups, except for IADL > 0 group. The association between frailty and PSIs was stronger in older adults, females, and individuals with a zero score of IADL than in younger adults, males, and those with a higher IADL score.

## Discussion

In this 3-year cohort study comprising 930 participants, we elucidated the characteristics of frailty and demonstrated a relationship between PSIs and frailty scores. To achieve this, various unsupervised machine-learning techniques were applied, including clustering, network analysis, and dimensionality reduction. Frailty was significantly associated with various clinical and laboratory variables, regardless of the different forms of frailty. Using unsupervised machine learning-based clustering methods, we classified participants into distinct frailty groups: robust, prefrail, and frail, demonstrating the robustness of our approach. Among PSIs, the UCLA loneliness scale emerged as the most representative indicator and showed a significant association with frailty. This association remained significant in subgroup analyses, underscoring the importance of psychosocial factors in the context of frailty.

Frailty is a clinical syndrome characterized by increased vulnerability to adverse health outcomes when exposed to stressors [24]. In 2001, the seminal frailty phenotype introduced by Fried et al. included five physical components to determine the frailty status [5]. Concurrently, the cumulative deficit model developed by Rockwood and Mitnitski encompasses 92 physical and psychosocial indices for classifying frailty [25]. At present, numerous frailty assessment tools are available, such as the Tilburg Frailty Indicator [26], comprising 25 indices, and the Groningen Frailty Indicator [19], consisting of 15 features, both of which integrate psychosocial dimensions. The current study used Fried’s criteria to examine the relationship between frailty and PSIs based on the cohort’s frailty assessment.

In the dataset used in this study, the distribution of frailty scores revealed that robust and prefrail participants constituted the majority, with frail individuals accounting for approximately 5% of the sample (Fig. S3B). This distribution was consistent with findings from other studies. For instance, a study applying Fried’s criteria to 3018 Chinese community-dwelling adults aged 65 years or older reported a distribution of 41% robust, 50% prefrail, and 8% frail individuals, similar to our study [27]. In a multicenter PROVIDE study, a UK-based study involving 132 sarcopenic and non-sarcopenic older adults, the distribution was 43% robust, 50% prefrail, and 7% frail [28]. Additionally, a meta-analysis of 21 cohorts of community-dwelling older adults using different frailty criteria found an average of 10.7% frail and 41.6% prefrail [29]. These findings underscore the consistency of frailty distribution across different populations and assessment tools. Consequently, our study can be interpreted and compared with previous studies, reinforcing the validity and relevance of the findings.

Furthermore, this study holds significant value from a medical informatics perspective, owing to the application of unsupervised machine learning techniques. Typically employed in genomic studies to identify hub genes, network analysis has been innovatively applied in the clinical research context to objectively and quantitatively identify the most relevant variables among clinical and laboratory measures. According to the network analysis, while the robust and prefrail groups showed high connectivity, the frail group exhibited lower connectivity (Fig. S5). This phenomenon likely resulted from the small sample size of the frail group, which affected the transformation of Pearson’s correlation coefficient to a *t*-value. Despite this limitation, highly connected hub variables were identified across all frailty status groups. When hierarchical clustering was performed using these hub variables, the three groups were visually and distinctly separable (Fig. 2).

The concept of frailty, acknowledged for its variability in definition [5, 19, 25, 26], was systematically operationalized in this study through the transformation of frailty scores ranging from 0 to 5 into multiple forms. These included a continuous linear variable from 0 to 5 (Cont), a binary variable distinguishing between robust and prefrail/frail individuals (Prefrail_bi), a binary variable that grouped robust and prefrail individuals together and compared them with frail individuals (Frail_bi), and a linear variable divided into three groups: robust, prefrail, and frail (tertile). Univariate associations between frailty and other indicators were evaluated using these distinct definitions. Through this approach, indicators that exhibited strong and consistent associations with frailty irrespective of the specific definition applied were identified (Figs. 3 and S8). This methodology ensured the robustness of the association between frailty and multiple indicators, thereby providing a comprehensive understanding of the relationship between frailty and its associated factors.

The relationship between frailty and clinical variables was analyzed to clarify the characteristics of frailty. The univariate associations between frailty and a range of clinical and laboratory variables, including age, education, alcohol consumption, ALM, and blood markers (TC, HDL, HbA1c, and Hb levels), as well as PSIs, suggest that frailty is a complex, multifactorial condition influenced by biological and physiological factors. By identifying these relationships, we can better understand the underlying mechanisms of frailty and develop targeted interventions to address its various dimensions. This comprehensive analysis reinforces the need for a holistic approach to assess and manage frailty considering the interplay between clinical, laboratory, and psychosocial variables.

Given the insufficient research on the mediating influences of frailty, social isolation, and loneliness on health pathwdays, there is a pressing need for additional studies [8]. Although numerous studies have investigated the association between social contact and frailty, a paucity of research directly compares the relationship between various psychosocial indices and frailty [14]. Moreover, as the older adults experiencing loneliness and social isolation continues to grow, understanding the association with frailty has become increasingly important [30, 31]. In this context, the present study is significant because it analyzed both the interrelationships among PSIs and their association with frailty (Fig. 4).

Each PSI incorporated in our study has repeatedly been shown to be associated with frailty [32–35]. However, their combined interplay has not been evaluated in a single cohort. Also, prior studies that implemented PSIs examined them in pairs or small subsets (Fig. S2) [36–47]. We therefore integrated all five instruments to capture distinct psychosocial domains—depressive symptoms, stress, social support, and loneliness (Figs. S9–S12).

Among the five PSIs indicating depressive symptoms, stress, loneliness, and social support, the UCLA loneliness scale demonstrated the strongest bridge centrality as verified by network analysis (Fig. S11). This finding is consistent with those of the previous studies. According to Liu et al., the UCLA 3-item loneliness scale can effectively identify older Chinese individuals experiencing depressive symptoms and serve as a quick community screening tool [48]. Additionally, in a large community sample of adults from Los Angeles County (*N* = 1000), those with lower levels of social support exhibited higher levels of stress and depressive symptoms [49]. These results highlight the significance of psychosocial factors, particularly loneliness, in the development of frailty. This emphasizes the need for comprehensive approaches, including psychosocial interventions, to manage and prevent frailty among older adults.

With a global increase in the older adults, the prevalence of frailty is also increasing, highlighting its growing importance [50, 51]. As the number of older adults increases, the population of frail individuals also grows, leading to substantial societal and healthcare burdens [52]. The substantial costs associated with this syndrome at both individual and societal levels are of particular concern in aging populations.

Building on this context, we explicitly incorporated age-stratified comparisons (≤ 65 vs > 65 years) to illuminate how frailty phenotypes and their psychosocial/clinical correlates differ across the life course (Fig. 3). The older group showed higher prevalence of overall frailty and each component. Notably, the between-group differences were largest for weakness and slowness, which likely contributed to the observed gap in overall frailty. PCC-based network analyses further revealed that loneliness (UCLA) served as greater hub centrality with age. Among frailty components, exhaustion and slowness demonstrated the strongest connections with PSIs—particularly depressive symptoms and perceived stress—suggesting that targeted attention to mood and stress management may be especially relevant for individuals exhibiting these features. In univariable analyses, associations between frailty features and psychological/clinical variables were more prominent in the > 65 group, indicating an age-related amplification of the coupling between frailty and psychosocial/clinical factors.

In this study, a subgroup analysis was performed to examine the association between frailty and PSIs, categorizing participants into groups above and below 65 years of age. Notably, the confidence intervals were narrower in the older age group (Fig. 5), suggesting that the impact of the PSI on frailty became more pronounced with advancing age. This highlights the critical importance of preventing frailty in older adults and emphasizes the need for psychosocial support strategies to mitigate the risk of frailty with age.

This study has several limitations. First, its cross-sectional design precludes causal inference between psychosocial indices and frailty. Second, psychosocial measures were self-reported, introducing potential information bias (e.g., recall and social-desirability effects) that may attenuate or inflate observed associations. Third, the frail subgroup constituted approximately 5% of the cohort, reducing statistical power and precision for within-group estimates and potentially limiting detection of modest effects. Future work incorporating longitudinal follow-up with repeated assessments, objective or clinician-rated measures, and adequately powered analyses of frail individuals will be important to corroborate and extend these findings. Fourth, in the network analyses, the clinical variables exhibited heterogeneous distributions (e.g., Gaussian, log-normal, and Bernoulli), making it difficult to adopt a single unified association metric that would be optimal for all variables. To address this issue, we mainly utilized Pearson’s correlation and additionally constructed networks using Spearman’s rank correlation and biweight midcorrelation; the nonparametric methods yielded network patterns broadly consistent with those derived from Pearson’s correlation (Fig. S13).

Despite these limitations, our study presents several key strengths. First, it delivers an integrative evaluation of psychosocial indices by comprehensively analyzing five complementary PSIs (PWI, CES-D, GDS, MSPSS, UCLA), an approach not previously applied in a single cohort (Fig. S2). Second, all PSIs exhibited a strong relationship with frailty status, with the lowest *p*-values (Table S3). Third, these relationships were robust across subgroups including age groups, supporting generalizability (Fig. 5). Collectively, the integrated PSI framework underscores the central role of psychosocial well-being in frailty and highlights actionable targets for assessment and intervention.

## Conclusions

This study demonstrated a strong association between frailty and PSIs, particularly loneliness, in a cohort of older Korean adults. Through a combination of unsupervised machine learning techniques and traditional regression analyses, we found that loneliness, as measured using the UCLA loneliness scale, serves as a central factor linking psychosocial variables to frailty. This relationship persisted across subgroup analyses, suggesting that psychosocial well-being is a critical component of frailty. These findings highlight the importance of incorporating psychosocial interventions into the management and prevention of frailty to improve health outcomes in aging populations.

## Supplementary information

Below is the link to the electronic supplementary material.

ESM 1 (DOCX 6.32 MB)

## Data Availability

The data and code supporting the findings of this study are available upon reasonable request and with permission from the corresponding authors.
