# Supplementary material for: The relationship between frailty status and psychosocial indices in older Korean adults
Source: GeroScience. 2025 Dec 17;48(5):7261–75. doi: 10.1007/s11357-025-02041-6 (PMC13601444; doi:10.1007/s11357-025-02041-6)
Supplement: Supplementary file 1 [file 11357_2025_2041_MOESM1_ESM.docx]

**The Relationship between Frailty Status and Psychosocial Indices in Korean Adults**

**(Supplementary Materials)**

Ji Young Kim^1,2^, Taesic Lee^3^, Sangbaek Koh^2^

^1^ Department of Family Medicine, Yonsei University Wonju College of Medicine, Wonju, Korea

^2^ Department of Preventive Medicine, Yonsei University Wonju College of Medicine, Wonju, Korea

^3^ Department of Convergence Medicine, Yonsei University Wonju College of Medicine, Wonju, Korea

Index

[**Supplementary Figure 1**. Flowchart of data collection	3](#_Toc213927374)

[**Supplementary Figure 2**. Prior literature mapping of pairwise combinations among the five PSIs.	4](#_Toc213927375)

[**Supplementary Figure 3**. Distribution of Frailty scale and its status categorized by cut-off value of 1 and 3 of frailty score.	5](#_Toc213927376)

[**Supplementary Figure 4**. Network analysis among robust, prefrail, and frail group with setting (A) 0.15, (B) 0.25, (C) 0.3 of PCC values as cut-off	6](#_Toc213927377)

[**Supplementary Figure 5.** The network patterns between clinical and laboratory variables and PSIs.	7](#_Toc213927378)

[**Supplementary Figure 6**. Hierarchical clustering and principal component analysis by variables that are significantly associated with frailty status	9](#_Toc213927379)

[**Supplementary Figure 7.** Identification of clinical and laboratory variables related to frailty status.	10](#_Toc213927380)

[**Supplementary Figure 8.** Univariate analysis of frailty and associated variables.	11](#_Toc213927381)

[**Supplementary Figure 9**. Correlation matrix and Network for five psychosocial indices.	12](#_Toc213927382)

[**Supplementary Figure 10**. General characteristics of the Aging-Cognitive cohort demonstrated by hierarchical clustering implementing PSI	13](#_Toc213927383)

[**Supplementary Figure 11**. General characteristics of the Aging-Cognitive cohort demonstrated by dimensionality reduction implementing PCA using PSI.	14](#_Toc213927384)

[**Supplementary Figure 12**. Network analysis among the five PSIs with PCC cut-off value 0.4.	15](#_Toc213927385)

[**Supplementary Figure 13**. Network analysis among robust, prefrail, and frail individuals constructed using Spearman correlation or biweight midcorrelation coefficients (cut-off = 0.30).	16](#_Toc213927386)

[**Supplementary Tables**	17](#_Toc213927387)

[**Supplementary Table 1.** Frailty score based on Fried’s criteria	17](#_Toc213927388)

[**Supplementary Table 2.** Psychosocial indices	18](#_Toc213927389)

[**Supplementary Table 3**. General characteristics of the Aging-Cognitive cohort according to status of frailty.	19](#_Toc213927390)

[**Supplementary Table 4**. Beta values and p-values for the univariate analysis of the relationship between frailty and associated variables. (A) continuous frailty score, (B) categorical frailty score	20](#_Toc213927391)

[**Supplementary Table 5**. Multivariate analysis covariates in previous studies	21](#_Toc213927392)

[**Supplementary Table 6.** Association between Eigenvalue of PSIs and Frailty	22](#_Toc213927393)

[**Supplementary Table 7.** Association between Loneliness (UCLA) and Frailty	23](#_Toc213927394)

**Supplementary Figures**

**Supplementary Figure 1**. Flowchart of data collection

## **Supplementary Figure 2**. Prior literature mapping of pairwise combinations among the five PSIs.

1. Jeon J, Kim S. Depression, stress and how they are related with health behaviors and metabolic syndrome among women over 40 years. J Korean Soc Matern Child Health 2012; 16(2): 263–73.

2. Bhang SY, Ha E, Park H, et al. Maternal Stress and Depressive Symptoms and Infant Development at Six Months: the Mothers and Children's Environmental Health (MOCEH) Prospective Study. J Korean Med Sci 2016; 31(6): 843–51.

3. Alhalaseh L, Kasasbeh F, Al-Bayati M, et al. Loneliness and Depression among Community Older Adults during the COVID-19 Pandemic: A cross-sectional study. Psychogeriatrics 2022; 22(4): 493–501.

4. Hughes ME, Waite LJ, Hawkley LC, Cacioppo JT. A Short Scale for Measuring Loneliness in Large Surveys: Results From Two Population-Based Studies. Res Aging 2004; 26(6): 655–72.

5. Cacioppo JT, Hughes ME, Waite LJ, Hawkley LC, Thisted RA. Loneliness as a specific risk factor for depressive symptoms: cross-sectional and longitudinal analyses. Psychol Aging 2006; 21(1): 140–51.

6. Cacioppo JT, Hawkley LC, Thisted RA. Perceived social isolation makes me sad: 5-year cross-lagged analyses of loneliness and depressive symptomatology in the Chicago Health, Aging, and Social Relations Study. Psychol Aging 2010; 25(2): 453–63.

7. Hawkley LC, Thisted RA, Masi CM, Cacioppo JT. Loneliness predicts increased blood pressure: 5-year cross-lagged analyses in middle-aged and older adults. Psychol Aging 2010; 25(1): 132–41.

8. Turner AD, Capuano AW, Wilson RS, Barnes LL. Depressive Symptoms and Cognitive Decline in Older African Americans: Two Scales and Their Factors. The American Journal of Geriatric Psychiatry 2015; 23(6): 568–78.

9. Qadir F, Haqqani S, Khalid A, Huma Z, Medhin G. A pilot study of depression among older people in Rawalpindi, Pakistan. BMC Research Notes 2014; 7(1): 409.

10. Grey I, Arora T, Thomas J, Saneh A, Tohme P, Abi-Habib R. The role of perceived social support on depression and sleep during the COVID-19 pandemic. Psychiatry Research 2020; 293: 113452.

11. Patra P, Alikari V, Fradelos EC, et al. Assessment of Depression in Elderly. Is Perceived Social Support Related? A Nursing Home Study : Depression and Social Support in Elderly. Adv Exp Med Biol 2017; 987: 139–50.

12. Gautam S, Poudel A, Khatry RA, Mishra R. The mediating role of perceived social support on loneliness and depression in community-dwelling Nepalese older adults. BMC Geriatrics 202413. Gale CR, Cooper C, Deary IJ, Aihie Sayer A. Psychological well-being and incident frailty in men and women: the English Longitudinal Study of Ageing. Psychological Medicine 2014; 44(4): 697–706.

14. Mezuk B, Edwards L, Lohman M, Choi M, Lapane K. Depression and frailty in later life: a synthetic review. International journal of geriatric psychiatry 2012; 27(9): 879–92.

15. Liu Y, Yang M, Zhao Y, et al. Social support mediates social frailty with anxiety and depression. BMC Pulmonary Medicine 2024; 24(1): 390.

16. Kojima G, Taniguchi Y, Aoyama R, Tanabe M. Associations between loneliness and physical frailty in community-dwelling older adults: A systematic review and meta-analysis. Ageing Research Reviews 2022; 81: 101705.

(A)

(B)

**Supplementary Figure 3**. Distribution of Frailty scale and its status categorized by cut-off value of 1 and 3 of frailty score.

(A)

(B)

(C)

**Supplementary Figure 4**. Network analysis among robust, prefrail, and frail group with setting (A) 0.15, (B) 0.25, (C) 0.3 of PCC values as cut-off

.(C)

**Supplementary Figure 5.** The network patterns between clinical and laboratory variables and PSIs.

(A) A Comparative analysis among network patterns according to frailty status (robust, prefrail, and frail groups, from left to right) was conducted. Each node represents a specific variable, with the color and size of the node indicating its connectivity. Small green nodes denote 0–4 edges, medium blue nodes represent 5–9 edges, and large red nodes signify 10 or more edges. The width of the edges corresponds to the absolute value of the *t*-statistic derived from Pearson correlation coefficients, using a threshold of 0.3. Red edges indicate positive correlations, while blue edges indicate negative correlations. Each network was established using samples specific to each frailty phenotype. (B) Histograms depict the variables with the highest degree of connectivity according to frailty status. Variables on the left show a high degree of connectivity, indicating strong associations with other variables, while those on the right exhibit fewer connections, reflecting weaker relationships.

The variables with top 50% number of edges in each frailty group were selected, which was 18 in robust, 17 in prefrail, and 20 in frail. The selected variables were included in the Venn diagram to obtain the intersecting variables from all three frailty groups.

Edu, education; HTN, hypertension; DM, diabetes mellitus; ALM, anti-lipid medication; BMI, body mass index; WC, waist circumference; SBP, systolic blood pressure; DBP, diastolic blood pressure; TC, total cholesterol; HDL, high-density lipoprotein; TG, triglyceride; Glc, glucose; HbA1c, hemoglobin A1c; BUN, blood urea nitrogen; Cr, creatinine; AST, aspartate aminotransferase; ALT, alanine aminotransferase; GGT, gamma-glutamyl transpeptidase; WBC, white blood cell; Hb, hemoglobin; MMSE, mini-mental state examination; CDR, clinical dementia rating; PWI, Psychological Well-Being Index; GDS, Geriatric Depression Scale; CES, Center for Epidemiologic Studies Depression Scale; MSPSS, Multidimensional Scale of Perceived Social Support; UCLA, University of California, Los Angeles Loneliness Scale; PSQI, Pittsburgh Sleep Quality Index; ADL, activities of daily living; IALD, instrumental activities of daily living; PSI: psychosocial index.

(A)

(B)

**Supplementary Figure 6**. Hierarchical clustering and principal component analysis by variables that are significantly associated with frailty status

## **Supplementary Figure 7.** Identification of clinical and laboratory variables related to frailty status.

(A) Univariate linear regression was performed between frailty and all variables. The results were normalized and assembled to form a figure. A dependent variable, frailty, was implanted either as a continuous variable with a value between 0 to 5 in the top row (LMcontinuous), or as a categorical variable where normal was converted to 1, prefrail to 2, and frail to 3 in the bottom row (LMcategorical). An independent variable was included in the figure only if its p-value was less than 0.1 in at least one linear model (LM). The color of each box indicates beta coefficient’s direction and strength; red indicates positive relationship whereas blue indicates negative relationship between an index and frailty status. Empty boxes with white color denote p-value larger than 0.1. P-value of each beta-coefficient is marked as asterisks. (B) Venn diagram of statistically significant variables according to different p-values. HTN; hypertension, DM; diabetes mellites, DLD; dyslipidemia, WC; waist circumference, TC; total cholesterol, HDL; high-density lipoprotein, Hb; hemoglobin, WBC; white blood cell, BUN; blood urea nitrogen, Cr; creatinine, ADL; activities of daily living, IADL; instrumental activities of daily living, CDR; Clinical Dementia Rating, MMSE; Mini‐Mental State Examination, PWI;

## **Supplementary Figure 8.** Univariate analysis of frailty and associated variables.

The circos plot illustrates the relationships between clinical and laboratory variables, PSIs, and frailty statuses. The nodes in the upper panel represent clinical and laboratory variables as well as PSIs, while the nodes in the lower panel denote the different forms of frailty status. “Cont,” “Prefrail_bi,” “Frail_bi,” and “Tertile” represent: (1) a continuous frailty score (ranging from 0–5), (2) a binary frailty score (dichotomized into robust and non-robust), (3) a binary frailty score (dichotomized into frail and non-frail), and (4) a tertile frailty score (categorized as robust, prefrail, and frail), respectively. The edges linking the upper and lower nodes signify statistically significant correlations (p < 0.05), determined by linear or logistic regression analysis depending on the specific frailty status model used. Edu, education; ALM, anti-lipid medication; TC, total cholesterol; HDL, high-density lipoprotein; HbA1c, hemoglobin A1c; Hb, hemoglobin; CDR, clinical dementia rating; MMSE, mini-mental state examination; PWI, Psychological Well-Being Index; GDS, Geriatric Depression Scale; CES, Center for Epidemiologic Studies Depression Scale; MSPSS, Multidimensional Scale of Perceived Social Support; UCLA, University of California, Los Angeles Loneliness Scale; PSQI, Pittsburgh Sleep Quality Index; IALD, instrumental activities of daily living; DM, diabetes mellitus; ADL, activities of daily living; HTN, hypertension; Glc, glucose; WBC, white blood cell; PSI, psychosocial index.

(A)

## **Supplementary Figure 9**. Correlation matrix and Network for five psychosocial indices.

Components located in diagonal indicate distributions of each psychosocial index. A scatter plot located in *i*^th^ column and *j*^th^ row illustrate correlation patterns between i^th^ and j^th^ diagonal features, their matched correlation coefficient was calculated by two methods, including Pearson and Spearman methods, which was located in *i*^th^ row and *j*^th^ column.

**Supplementary Figure 10**. General characteristics of the Aging-Cognitive cohort demonstrated by hierarchical clustering implementing PSI

**Supplementary Figure 11**. General characteristics of the Aging-Cognitive cohort demonstrated by dimensionality reduction implementing PCA using PSI.

**Supplementary Figure 12**. Network analysis among the five PSIs with PCC cut-off value 0.4.

(A) Spearman correlation

(B) Biweight midcorrelation

**Supplementary Figure 13**. Network analysis among robust, prefrail, and frail individuals constructed using Spearman correlation or biweight midcorrelation coefficients (cut-off = 0.30).

**Supplementary Tables**

**Supplementary Table 1.** Frailty score based on Fried’s criteria

| Criteria | Condition |
| --- | --- |
| 1. Weight loss | unintentional weight loss more than 4.5kg or 5% of one’s weight in recent one year |
| 2. Weakness | grip strength less than 26kg in men and 18kg in women (highest value among three measurements) |
| 3. Exhaustion | self-reported in the scale of 1 to 5. Over the past one month, participants were categorized as exhausted if they consistently (1) or predominantly (2) reported feeling tired, and as not exhausted if they occasionally (3) or rarely (4) felt tired or not at all (5) |
| 4. Slowness | 4.82 seconds or more to walk 4 meters (better value among two measurements) |
| 5. Low activity | ≤ 494.65 kcal for men and ≤ 283.50 kcal for women in average one week |

## **Supplementary Table 2.** Psychosocial indices

| Category | Character | Condition |
| --- | --- | --- |
| Psychological | Stress | Psychological Well-Being Index (PWI) |
|  | Depression | Center for Epidemiologic Studies Depression Scale (CES) |
|  | Depression | Geriatric Depression Scale (GDS) |
| Social | Loneliness | University of California, Los Angeles Loneliness Scale (UCLA) |
|  | Social support | Multidimensional Scale of Perceived Social Support (MSPSS) |

## **Supplementary Table 3**. General characteristics of the Aging-Cognitive cohort according to status of frailty.

|  | **Total** | **Robust** | **Pre-frail** | **Frail** | **P-value** |
| --- | --- | --- | --- | --- | --- |
| Age | 67.8 ± 0.21 | 66.8 ± 0.29 | 68.4 ± 0.3 | 72 ± 0.79 | <0.001 |
| Sex | 576 (61.9) | 264 (59.5) | 279 (63.6) | 33 (70.2) | 0.222 |
| Edu | 10.6 ± 0.14 | 11.4 ± 0.19 | 10.1 ± 0.21 | 7.7 ± 0.56 | <0.001 |
| Marry | 816 (87.7) | 400 (90.1) | 379 (86.3) | 37 (78.7) | 0.036 |
| Alcohol | 308 (33.1) | 168 (37.8) | 132 (30.1) | 8 (17) | 0.003 |
| Smoking | 45 (4.8) | 19 (4.3) | 25 (5.7) | 1 (2.1) | 0.417 |
| HTN | 420 (45.2) | 188 (42.3) | 205 (46.7) | 27 (57.4) | 0.095 |
| DM | 376 (40.4) | 160 (36) | 191 (43.5) | 25 (53.2) | 0.015 |
| ALM | 184 (19.8) | 67 (15.1) | 99 (22.6) | 18 (38.3) | <0.001 |
| BMI | 24.6 ± 0.1 | 24.6 ± 0.14 | 24.6 ± 0.14 | 24.8 ± 0.51 | 0.703 |
| WC | 85.7 ± 0.28 | 85.2 ± 0.41 | 86.2 ± 0.41 | 86.2 ± 1.28 | 0.085 |
| SBP | 130.1 ± 1.42 | 128.8 ± 0.74 | 132 ± 2.89 | 125.6 ± 2.55 | 0.623 |
| DBP | 78.4 ± 1.43 | 77.7 ± 0.44 | 79.8 ± 3 | 72.2 ± 1.44 | 0.976 |
| TC | 184.2 ± 1.33 | 187.4 ± 1.91 | 182.7 ± 1.94 | 168.2 ± 5.57 | 0.003 |
| HDL | 53.3 ± 0.45 | 54.3 ± 0.65 | 52.9 ± 0.65 | 48 ± 1.74 | 0.005 |
| TG | 126.6 ± 2.15 | 125.6 ± 3.03 | 127.3 ± 3.24 | 128.8 ± 8.59 | 0.643 |
| Glc | 99 ± 0.63 | 97.8 ± 0.88 | 99.9 ± 0.89 | 102.3 ± 4.36 | 0.045 |
| HbA1c | 6 ± 0.02 | 5.9 ± 0.03 | 6 ± 0.03 | 6.3 ± 0.16 | <0.001 |
| BUN | 17.4 ± 0.2 | 17.2 ± 0.23 | 17.4 ± 0.29 | 19.2 ± 1.67 | 0.115 |
| Cr | 0.8 ± 0.01 | 0.8 ± 0.01 | 0.8 ± 0.02 | 0.9 ± 0.09 | 0.514 |
| AST | 26.5 ± 0.36 | 26.2 ± 0.43 | 26.9 ± 0.61 | 26.2 ± 1.45 | 0.523 |
| ALT | 23.3 ± 0.43 | 23.3 ± 0.59 | 23.3 ± 0.62 | 23.7 ± 2.71 | 0.938 |
| GGT | 18.4 ± 0.83 | 17.3 ± 0.83 | 19.4 ± 1.51 | 19 ± 3.6 | 0.25 |
| WBC | 5.5 ± 0.05 | 5.4 ± 0.07 | 5.4 ± 0.07 | 6 ± 0.26 | 0.12 |
| Hb | 13.6 ± 0.04 | 13.8 ± 0.06 | 13.5 ± 0.06 | 12.8 ± 0.16 | <0.001 |
| MMSE | 27.3 ± 0.08 | 27.6 ± 0.1 | 27.2 ± 0.12 | 25.9 ± 0.48 | <0.001 |
| CDR | 0 ± 0 | 0 ± 0 | 0 ± 0 | 0.1 ± 0.03 | <0.001 |
| PWI | 16.5 ± 0.27 | 14.2 ± 0.31 | 17.9 ± 0.43 | 24.4 ± 1.29 | <0.001 |
| GDS | 9.5 ± 0.22 | 7.6 ± 0.25 | 10.7 ± 0.34 | 16.3 ± 1.01 | <0.001 |
| CES | 9.5 ± 0.3 | 7.4 ± 0.36 | 10.7 ± 0.45 | 18.6 ± 1.62 | <0.001 |
| MSPSS | 47.7 ± 0.28 | 48.4 ± 0.39 | 47.3 ± 0.41 | 44 ± 1.46 | <0.001 |
| UCLA | 36.3 ± 0.29 | 34.6 ± 0.37 | 37.2 ± 0.44 | 43.4 ± 1.59 | <0.001 |
| PSQI | 5.7 ± 0.12 | 5.3 ± 0.16 | 5.9 ± 0.17 | 8.1 ± 0.68 | <0.001 |
| ADL | 20 ± 0.01 | 20 ± 0 | 20 ± 0.01 | 19.8 ± 0.13 | <0.001 |
| IADL | 0 ± 0.01 | 0 ± 0.01 | 0.1 ± 0.01 | 0.1 ± 0.05 | 0.002 |

**Supplementary Table 4**. Beta values and p-values for the univariate analysis of the relationship between frailty and associated variables. (A) continuous frailty score, (B) categorical frailty score

(A)	(B)

| Feature | Beta coefficient | p-value |
| --- | --- | --- |
| Age | 0.19864905 | 8.25E-12 |
| Sex | 0.05733789 | 5.12E-02 |
| Edu | -0.1946824 | 2.17E-11 |
| Marry | -0.088869 | 2.47E-03 |
| Alcohol | -0.1184309 | 5.31E-05 |
| Smoking | 0.02854557 | 3.32E-01 |
| HTN | 0.07740788 | 8.42E-03 |
| ALM | 0.13968621 | 1.80E-06 |
| DM | 0.08845739 | 2.59E-03 |
| SBP | 0.01482284 | 6.15E-01 |
| DBP | 0.00060251 | 9.84E-01 |
| BMI | 0.00492592 | 8.67E-01 |
| WC | 0.04600202 | 1.18E-01 |
| TC | -0.0975795 | 8.84E-04 |
| HDL | -0.0788157 | 7.30E-03 |
| TG | 0.01223908 | 6.78E-01 |
| Glc | 0.07405999 | 1.17E-02 |
| HbA1c | 0.12551047 | 1.82E-05 |
| BUN | 0.06747111 | 2.17E-02 |
| Cr | 0.04935095 | 9.34E-02 |
| AST | 0.00693686 | 8.14E-01 |
| ALT | -0.0050399 | 8.64E-01 |
| GGT | 0.03165004 | 2.82E-01 |
| WBC | 0.06685028 | 2.29E-02 |
| Hb | -0.1591837 | 4.99E-08 |
| CDR | 0.13698616 | 2.85E-06 |
| MMSE | -0.1615974 | 3.10E-08 |
| PWI | 0.31540498 | 1.72E-28 |
| GDS | 0.32366143 | 5.11E-30 |
| CES | 0.27431541 | 1.48E-21 |
| MSPSS | -0.1057861 | 3.13E-04 |
| UCLA | 0.21260992 | 2.47E-13 |
| PSQI | 0.15910756 | 5.07E-08 |
| ADL | -0.1453345 | 6.67E-07 |
| IADL | 0.11205085 | 1.32E-04 |

| Feature | Beta coefficient | p-value |
| --- | --- | --- |
| Age | 0.111967855 | 4.92E-09 |
| Sex | 0.032978936 | 0.08753 |
| Edu | -0.12295565 | 1.22E-10 |
| Marry | -0.04810651 | 0.012613 |
| Alcohol | -0.06519573 | 0.000711 |
| Smoking | 0.006064923 | 0.753542 |
| HTN | 0.039540782 | 0.040439 |
| ALM | 0.079791653 | 3.33E-05 |
| DM | 0.055918198 | 0.003716 |
| SBP | 0.009485444 | 0.623379 |
| DBP | 0.000574467 | 0.976276 |
| BMI | 0.00737508 | 0.702608 |
| WC | 0.033229078 | 0.08515 |
| TC | -0.05761258 | 0.002793 |
| HDL | -0.05402771 | 0.005066 |
| TG | 0.008945863 | 0.643271 |
| Glc | 0.038695302 | 0.044929 |
| HbA1c | 0.073834025 | 0.000124 |
| BUN | 0.030402311 | 0.11528 |
| Cr | 0.012603147 | 0.514075 |
| AST | 0.012349723 | 0.522574 |
| ALT | 0.001512916 | 0.937574 |
| GGT | 0.022226443 | 0.249727 |
| WBC | 0.029988659 | 0.120318 |
| Hb | -0.09425841 | 9.05E-07 |
| CDR | 0.075420274 | 8.84E-05 |
| MMSE | -0.09206006 | 1.62E-06 |
| PWI | 0.181512579 | 5.84E-22 |
| GDS | 0.190064565 | 4.91E-24 |
| CES | 0.159912021 | 3.52E-17 |
| MSPSS | -0.06390007 | 0.000914 |
| UCLA | 0.130261339 | 8.93E-12 |
| PSQI | 0.089662044 | 3.03E-06 |
| ADL | -0.07445888 | 0.000109 |
| IADL | 0.058638772 | 0.002341 |

**Supplementary Table 5**. Multivariate analysis covariates in previous studies

|  | Chongya Zhai et al. (2024) | B. Davies et al. (2022) | CS Blaum et al. (2005) | Liu et al. (2023) | Yang et al. (2023) |
| --- | --- | --- | --- | --- | --- |
| Age | Model 1 | Model 1 | Model 1 | Model 1 | Model 1 |
| Sex | Model 1 | Model 1 |  | Model 1 | Model 1 |
| Race | Model 1 |  | Model 1 | Model 1 | Model 1 |
| marital status |  |  |  | Model 1 | Model 1 |
| BMI | Model 2 |  |  |  | Model 2 |
| education | Model 2 |  | Model 1 | Model 1 | Model 1 |
| alcohol | Model 2 |  |  | Model 2 |  |
| smoking | Model 2 |  |  | Model 2 |  |
| income | Model 2 |  |  | Model 1 | Model 1 |
| leisure-time physical activity | Model 2 |  |  |  |  |
| sarcopenia |  | Model 2 |  |  |  |
| DM | Model 3 | Model 3 | Model 2 |  | Model 3 |
| Dyslipidemia | Model 3 |  |  |  | Model 3 |
| HTN | Model 3 |  |  |  | Model 3 |
| Malignancy | Model 3 | Model 3 |  |  |  |
| Coronary arterty disease |  | Model 3 | Model 2 |  | Model 3 |
| Heart failure |  | Model 3 | Model 2 |  |  |
| Peripheral vascular disease |  | Model 3 |  |  |  |
| Cerebrovascular disease |  | Model 3 |  |  | Model 3 |
| Dementia |  | Model 3 |  |  |  |
| Chronic pulmonary disease |  | Model 3 |  |  |  |
| Peptic ulcer disease |  | Model 3 |  |  |  |
| Liver disease |  | Model 3 |  |  |  |
| Hemiplegia or paraplegia |  | Model 3 |  |  |  |
| Renal disease |  | Model 3 |  |  | Model 3 |
| AIDS |  | Model 3 |  |  |  |
| CRP |  |  | Model 3 |  |  |
| serum carotinoids |  |  | Model 3 |  |  |
| total energy intake |  |  |  | Model 3 |  |
| healthy eating index |  |  |  | Model 3 |  |
| Alb |  |  |  |  | Model 2 |
| Hb |  |  |  |  | Model 2 |
| UA |  |  |  |  | Model 2 |
| WBC |  |  |  |  | Model 2 |
| ALT |  |  |  |  | Model 2 |
| AST |  |  |  |  | Model 2 |
| Cr |  |  |  |  | Model 2 |

Abbreviations: BMI, body mass index; DM, diabetes mellitus; HTN, hypertension; AIDS, acquired immunodeficiency syndrome; CRP, C-reactive protein; Alb, albumin; Hb, hemoglobin; UA, uric acid; WBC, white blood cell; ALT, alanine transaminase; AST, aspartate aminotransferase; Cr, creatinine.

**Supplementary Table 6.** Association between Eigenvalue of PSIs and Frailty

|  |  | Beta | SE | p-value |
| --- | --- | --- | --- | --- |
| Linear | Model1 | -0.020 | 0.002 | 4.21e-24 |
|  | Model2 | -0.019 | 0.002 | 6.22e-20 |
|  | Model3 | -0.018 | 0.002 | 8.56e-19 |

Model1: age, sex

Model2: education, marital status, alcohol, smoking, BMI

Model3: disease (HTN, DM, HLD), lab (TC, HDL, Hb)

**Supplementary Table 7.** Association between Loneliness (UCLA) and Frailty

|  |  | Beta | SE | p-value |
| --- | --- | --- | --- | --- |
| Linear | Model1 | 0.021 | 0.003 | 9.23e-11 |
|  | Model2 | 0.019 | 0.003 | 5.5e-09 |
|  | Model3 | 0.018 | 0.003 | 2.27e-08 |
|  |  | OR (95% CI) | | p-value |
| Logistic | Model1 | 1.04 (1.023 - 1.056) | | 1.67e-06 |
|  | Model2 | 1.036 (1.02 - 1.053) | | 1.65e-05 |
|  | Model3 | 1.035 (1.018 - 1.052) | | 3.56e-05 |

Model1: age, sex

Model2: education, marital status, alcohol, smoking, BMI

Model3: disease (HTN, DM, HLD), lab (TC, HDL, Hb)
